# Vitamin K for Vascular Calcification in Kidney Patients: Still Alive and Kicking, but Still a Lot to Learn

**DOI:** 10.3390/nu16121798

**Published:** 2024-06-07

**Authors:** Ioannis Eleftherios Neofytou, Aikaterini Stamou, Antonia Demopoulos, Stefanos Roumeliotis, Pantelis Zebekakis, Vassilios Liakopoulos, Eleni Stamellou, Evangelia Dounousi

**Affiliations:** 1 2nd Department of Nephrology, AHEPA Hospital, Medical School, Aristotle University of Thessaloniki, 54636 Thessaloniki, Greece; john_neofytou_@hotmail.com (I.E.N.); katerina_stms@yahoo.gr (A.S.); antoniaed@auth.gr (A.D.); vliak@auth.gr (V.L.); 2 1st Department of Internal Medicine, AHEPA Hospital, Medical School, Aristotle University of Thessaloniki, 54636 Thessaloniki, Greece; pzempeka@auth.gr; 3 Department of Nephrology, Faculty of Medicine, School of Health Sciences, University of Ioannina, 45110 Ioannina, Greece; stamellou.eleni@googlemail.com (E.S.); edounous@uoi.gr (E.D.); 4 Division of Nephrology and Clinical Immunology, RWTH Aachen University, 52062 Aachen, Germany

**Keywords:** CKD, vascular calcification, dp-ucMGP, PIVKA-II, vitamin K, menaquinone-7

## Abstract

Patients with chronic kidney disease (CKD) suffer disproportionately from a high burden of cardiovascular disease, which, despite recent scientific advances, remains partly understood. Vascular calcification (VC) is the result of an ongoing process of misplaced calcium in the inner and medial layers of the arteries, which has emerged as a critical contributor to cardiovascular events in CKD. Beyond its established role in blood clotting and bone health, vitamin K appears crucial in regulating VC via vitamin K-dependent proteins (VKDPs). Among these, the matrix Gla protein (MGP) serves as both a potent inhibitor of VC and a valuable biomarker (in its inactive form) for reflecting circulating vitamin K levels. CKD patients, especially in advanced stages, often present with vitamin K deficiency due to dietary restrictions, medications, and impaired intestinal absorption in the uremic environment. Epidemiological studies confirm a strong association between vitamin K levels, inactive MGP, and increased CVD risk across CKD stages. Based on the promising results of pre-clinical data, an increasing number of clinical trials have investigated the potential benefits of vitamin K supplementation to prevent, delay, or even reverse VC, but the results have remained inconsistent.

## 1. Introduction

Chronic kidney disease (CKD) dramatically increases the risk of cardiovascular disease (CVD); this risk is rising in parallel with the deterioration of kidney function [1,2]. Emerging factors linked to kidney dysfunction, such as inflammation, oxidative stress, and most notably vascular calcification (VC), are gaining recognition for their critical role in CVD and reduced patient survival [3,4,5,6].

**Figure 1:**
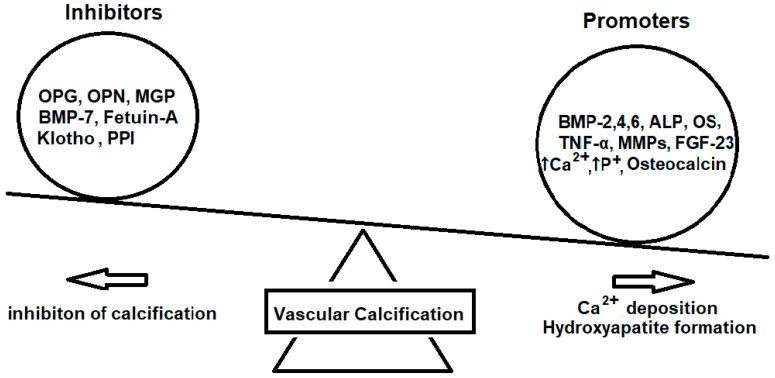
The disrupted balance between promoters and inhibitors of vascular calcification that inclines towards the deposition of calcium and hydroxyapatite formation on the vessels. (ALP: Alkaline phosphate, BMP: Bone Morphogenetic Proteins, FGF-23: Fibroblast Growth Factor 23, MGP: Matrix Gla Protein, MMPs: Matrix Metalloproteinases, OPG: Osteoprotegerin, OPN: Osteopontin, OS: Oxidative Stress, PPI: Pyrophosphate Ions, TNF-α: Tumor Necrosis Factor alpha).

VC occurs early in CKD and increases progressively, as kidney function declines. CKD patients exhibit all four types of VC: intimal and medial calcification in arteries, heart valve calcification, and calciphylaxis, which is more disease-specific for dialysis (Ca^2+^ deposits in small arteries leading to the necrotization of skin and fat tissue, causing painful ulcers) [7,8]. Each type significantly and independently increases the risk of CV morbidity and mortality in these patients [9,10,11]. Microcalcification of the arterial wall is found 45 times more often in CKD patients compared to age–gender-matched controls from the general population, whereas at pre-dialysis CKD, the prevalence of intimal and medial calcification ranges from 50 to 90%, and after 5 years on dialysis, heart valve calcification is reported in more than 80%. The balance between activators and inhibitors of VC (Figure 1) is severely deranged in CKD and especially ESKD, due to the pronounced upregulation of activators and suppression of the function and/or reduction in circulating inhibitors by the uremic environment and by dialysis-related factors [12]. Notably, even after years of undergoing hemodialysis (HD), 10–20% of patients do not present VC because they are protected from the naturally occurring defense inhibitors of VC [13]. In healthy subjects, VC is a degenerative process, which increases with advanced aging. Under this perspective, CKD might be considered as a clinical model of premature and abnormally increased aging, which leads to arterial aging as well. This early arterial aging in both micro- and macrovasculature in uremia is driven by several factors, including endothelial dysfunction, inflammation, oxidative stress, genomic instability, cell senescence, uremic toxins accumulation, attrition of telomeres, mitochondrial dysfunction, and metabolic disorders.

Among inhibitor proteins, the matrix Gla protein (MGP) stands out as the most powerful and clinically important [9,14]. Although it was first discovered in bone tissue and was considered to be a bone-regulating protein back in 1987 [15], MGP has emerged as an important key player in VC when Luo et al. in 1997 observed rapid arterial calcification and premature death due to rupture of the aorta (within weeks) in knock-out animal models for MGP rats [16]. Following this landmark study, subsequent research demonstrated the critical role of MGP in the pathogenesis of VC [14,17].

MGP is a small 84 amino acid protein, containing 3 serine and 5 glutamate residues that is expressed in bone, arteries, heart, cartilage, and kidneys. Once activated, MGP delays the progression or even regresses VC, in some cases, by directly scavenging free, reactive calcium ions, phosphorus ions, and hydroxyapatite crystals from the arterial wall and then disposes them into circulation with a vacuum-like mechanism; and indirectly, by downregulating the promoter of VC bone morphogenetic protein-2 (BMP-2). These actions require the activation of MGP, which is a two-step process that is heavily dependent on the availability of vitamin K [18]. Vitamin K acts as an essential co-factor for γ-carboxylation of glutamate and the sequential phosphorylation of serine residues to transform into the fully inactive form (dephosphorylated, uncarboxylated dp-ucMGP) of MGP. Therefore, MGP exists in four forms, dp-ucMGP, the partially inactive carboxylated dephosphorylated and uncarboxylated form, and the fully active form. Experimental data showed that dp-ucMGP aggregates at sites of calcification, and clinical studies have coherently shown a strong association with atherosclerosis, arterial stiffness, CVD, and mortality in CKD and HD patients [19]. MGP belongs to a larger family of 17 vitamin K-dependent protein-VKDPs, including prothrombin, proteins C and S, and osteocalcin [20,21], which all need vitamin K to become activated and regulate hemostasis and bone and artery health [22]. Thus, the fully inactivated form of MGP, circulating dp-ucMGP, reflects poor vitamin K status and is a strong, independent predictor of VC and CVD in ESKD patients [23]. Moreover, in pre-dialysis CKD, plasma dp-ucMGP levels progressively increase in accordance with the deterioration of the glomerular filtration rate (GFR), indicating uremia as a state of pronounced vitamin K deficiency [24,25].

This review aims to address the unresolved issues of vitamin K dietary recommended dose for CKD patients, which biomarker reflects vitamin K deficiency, and present a critical appraisal of controlled clinical trials examining vitamin K supplementation in CV outcomes. Moreover, since the existing data are controversial due to methodological flaws of the trials published so far, we also provide suggestions for the design of future controlled trials in this field.

## 2. Vitamin K Biomarkers

In both research and clinical practice, direct measurement of vitamin K levels in the blood has certain limitations [26]. First, circulating vitamin K represents only a small fraction of total body stores and varies greatly depending on recent dietary intake [27]. Secondly, although phylloquinone (vitamin K1) is relatively easy to measure, menaquinone [28] (vitamin K2) is a small particle that can only be detected in very high concentrations [29]. Third, these measurements may not detect subclinical vitamin K deficiency, which can negatively impact health before prothrombin time (PT) prolongation [30,31,32]. Finally, the optimal biomarker assessing vitamin K status has to reflect both the status and the clinical importance of this vitamin. Therefore, the approach of measuring VKDPs as biomarkers could enable a more sensitive and reliable assessment of functional vitamin K status.

The collective measurement of the inactive forms of VKDPs emerges as a rational alternative to use as biomarkers for vitamin K, due to their direct dependence on vitamin K for activation and function [33]. VKDPs provide insight into vitamin K status and implicate the consequences of its insufficiency for protein activation [34]. Initially, undercarboxylated osteocalcin (OC) was used as a biomarker, but it is mainly concentrated in bone tissue and its sensitivity is influenced by vitamin D and PTH levels (which are often disturbed in advanced CKD) [35,36]. Therefore, OC showed limited benefit, particularly in the CKD population, and is not sufficiently representative of other vitamin K properties, such as blood coagulation and VC. As research progressed, two additional VKDPs gained prominence as valuable biomarkers: dp-ucMGP and protein induced in the absence of vitamin K or antagonism factor II (PIVKA-II) [37]. Dp-ucMGP accurately represents circulating vitamin K and has been confirmed by several studies to be strongly associated with VC in the CKD and ESKD population [9,38], while PIVKA-II reflects the vitamin K liver status of prothrombin, a key coagulation factor [39,40]. Studies have consistently shown a strong inverse correlation between circulating dp-ucMGP and PIVKA-II levels and plasma vitamin K concentration in various populations, including healthy and CKD subjects [39,41,42].

Soon after Schurgers et al. coined a novel sandwich antibody detection method for measuring circulating dp-ucMGP, this marker gradually emerged as a well-studied and reliable circulating biomarker of vitamin K status. Recent research has demonstrated its significant association with various markers of arterial calcification and stiffness-carotid intima-media thickness, pulse-wave velocity (PWV), and coronary calcification score-CVD and overall mortality in CKD patients across stages [42]. A cohort study of 107 renal patients with CKD stages 2–5 showed elevated dp-ucMGP levels, which were significantly associated with the progression of renal failure, degree of aortic calcification, and all-cause mortality [43]. In a similar study of 137 CKD patients at various stages, Puzantian et al. observed that dp-ucMGP levels rose with worsening CKD stage and were an independent risk factor for worsening carotid-femoral pulse wave velocity (PWV) [44]. Two independent studies, each involving 160 and 50 HD patients, confirmed an association between dp-ucMGP levels and abdominal aortic calcification assessed by spiral computed tomography (CT). Furthermore, in one of the two studies, dp-ucMGP levels and duration since starting hemodialysis were positively correlated [45,46]. Schlieper et al. showed that increased dp-ucMGP concentration was associated with all-cause mortality in HD patients [47]. Increased mortality associated with high dp-ucMGP levels was also reported in a large study of patients with stage 5 CKD, who were followed for up to 42 months. Interestingly, the correlation in this study was independent of the VC status of the patients [48]. In agreement with these findings, a small observational study with a long 7-year follow-up showed that increased circulating dp-ucMGP was a strong, independent predictor of death (hazard ratio-HR = 2.63, 95% confidence interval-CI = 1.17–5.94), CV mortality (HR = 2.82, 95% CI = 1.07–7.49), and CKD progression to ESKD or at least a 30% decline in GFR (HR = 4.02, 95% CI = 1.20–13.46) [24]. Finally, a large longitudinal study of 518 kidney transplant recipients over a median period of 9.8 years concluded that dp-ucMGP levels served as an independent predictor of both all-cause mortality and graft failure [49].

Protein induced in the vitamin K absence or antagonism-factor II (PIVKA-II), first reported by Liebman et al. in 1984, serves as a marker for the vitamin K status of coagulation-relevant proteins [50]. Vitamin K is necessary for the γ-carboxylation of the glutamic acid residues in these proteins [51]. Elevated PIVKA-II levels, usually undetectable in healthy individuals, indicate vitamin K deficiency, which results in undercarboxylation of prothrombin (factor II), an inactive form that cannot properly participate in blood clotting [32,52]. It acts as a more sensitive indicator of vitamin K deficiency compared to prolonged PT [53]. Clinically, PIVKA-II is used as a biomarker of vitamin K levels in newborns [54], as a diagnostic marker for hepatocellular carcinoma (in combination with α-fetoprotein) [55] and as a monitor for patients receiving vitamin K antagonists [56]. The benefit of the PIVKA-II measurement is also being studied in CKD patients [57,58]. 

PIVKA levels inversely correlate with dietary vitamin K intake [40]. In one study, a gradual increase in detectable PIVKA-II levels was observed as CKD progressed. While PIVKA-II was rarely detected in stage 3 CKD, it was more common in stage 4 and often detected in stage 5 CKD patients. This trend was further supported by two studies of HD patients in which 83% to 97% of participants had abnormal PIVKA-II levels [59,60]. However, a Polish study of HD patients reported PIVKA-II levels similar to those of healthy volunteers. It is noteworthy that this study used a different assay for the PIVKA-II measurement compared to the remaining studies [61]. Additionally, a study of 28 patients undergoing continuous ambulatory peritoneal dialysis (CAPD) found that half of the participants had abnormal PIVKA-II levels [62]. 

Some studies have examined the correlation between PIVKA-II and clinical outcomes in CKD patients, but data are currently limited. A study of 44 HD patients confirmed the high prevalence of abnormal PIVKA-II levels (91%) in end-stage CKD and revealed a significant association between elevated PIVKA-II and coronary artery disease (CAD) [58]. This finding suggests that PIVKA-II may be a potential marker for increased cardiovascular risk. Additionally, a recent study of 69 HD patients contributed to the investigation of the association between PIVKA-II and the coronary artery calcification (CAC) score. While the study found a positive association between the PIVKA-II and CAC score in diabetic HD patients, this correlation was not statistically significant in non-diabetic HD patients [63]. These findings require further investigation in larger studies to confirm the potential role of PIVKA-II as a biomarker for cardiovascular complications in CKD. Both studies involved relatively few participants, highlighting the need for further research with larger and more diverse patient populations. 

In summary, direct measurement of vitamin K is impractical and does not accurately reflect the true functional vitamin K status. Hence, VKDPs, such as PIVKA-II and dp-ucMGP, serve as better biomarkers; they represent different aspects of vitamin K activity in the body. Studies have shown a weak correlation between PIVKA-II and dp-ucMGP, supporting their distinct roles [64]. The dp-ucMGP acts as a marker of circulating vitamin K and is tightly associated with VC. In contrast, PIVKA-II reflects hepatic vitamin K status and may be a more sensitive and earlier indicator of impaired coagulation, a process heavily reliant on vitamin K. Notably, PIVKA-II levels are not affected by renal function and the cholesterol-triglycerides levels in contrast to vitamin K [58]. In several studies, abnormal levels of both PIVKA-II and dp-ucMGP have been observed in CKD patients. While dp-ucMGP has a well-established association with CVD, the link between PIVKA-II and CVD remains under investigation. Further research is needed to explore the potential role of PIVKA-II as a biomarker for cardiovascular complications in CKD.

## 3. Clinical Aspects of Vitamin K Supplementation

### 3.1. What Is the Actual Recommended Daily Intake?

Vitamin K, a fat-soluble essential micronutrient obtained exclusively through diet, plays a vital role in three essential bodily functions: blood clotting, promoting skeletal health through osteocalcin activation, and preventing VC. In newborns, vitamin K supplementation is a worldwide practice for the control of hemostasis [65,66]. However, its importance in adults is underestimated because, in contrast to the established recommended daily dose for vitamin K1 (women: 90 µg/day, men: 120 µg/day), there is currently no unanimously accepted dosage for vitamin K2 [7,32,67]. Moreover, these recommended doses of vitamin K aim to prevent evident coagulation abnormalities, and the daily dosage required for the carboxylation of VKDPs has not been thoroughly studied [68]. In the general population, a relatively balanced diet ensures adequate vitamin K intake and deficiency is only occasionally reported [26,69]. Additionally, vitamin K toxicity due to excessive consumption has not yet been reported, making it a safe choice and therefore does not set an upper limit for consumption [70,71,72]. However, vitamin K status in CKD patients differs from the rest of the population [73]. The current recommended dietary intake (RDI) refers only to vitamin K1 and aims to prevent hemostasis disorders. However, there are currently no reports on the recommended intake of vitamin K2 for preventing VC nor any specific recommendations for patients with heavy CV burden, such as diabetic, CKD, and ESKD patients.

### 3.2. Vitamin K Status in Kidney Disease

In CKD patients, several factors contribute to the high prevalence of vitamin K deficiency [74]. In particular, in advanced stages, dietary restrictions to limit potassium, phosphorus, and protein intake inadvertently result in the exclusion of important sources of vitamin K, such as dark leafy vegetables and dairy products [75]. As a result, more than half of CKD patients report consuming less than the recommended daily vitamin K intake [59]. While this amount may be sufficient to avoid PT prolongation, it may lead to subclinical vitamin K deficiency and its long-term consequences [69]. In particular, several VKDPs would fail due to the absence of vitamin K, unable to be fully carboxylated or function properly, resulting in an inability to maintain bone health or to prevent VC [23,76].

**Figure 2:**
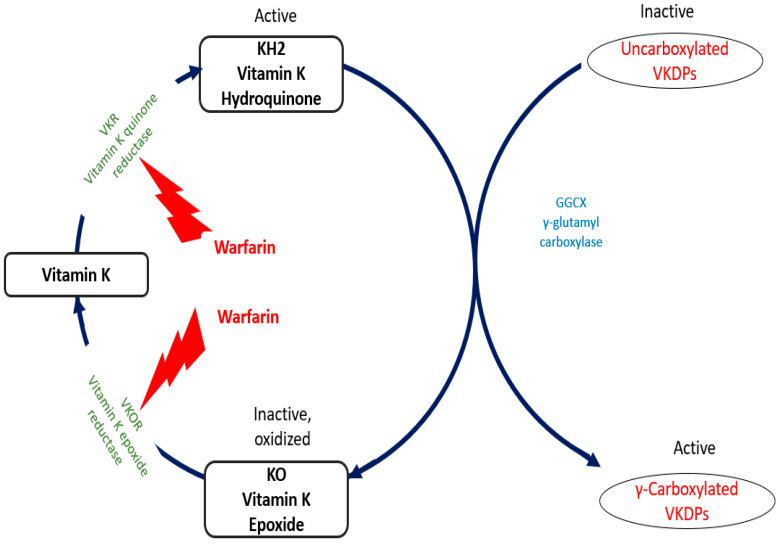
GGCX (γ-glutamyl carboxylase) is a vitamin K-dependent enzyme responsible for γ-carboxylation of the inactive to active VKDPs. During this process, KH2 is converted to KO (vitamin K epoxide). KO is converted to vitamin K by VKOR (vitamin K epoxide reductase) and then vitamin K is converted to KH2 by VKR (Vitamin K reductase). Warfarin inhibits the function of both VKOR and VKR.

In addition, CKD patients are prescribed a number of medications that interfere with the absorption and effects of vitamin K [77]. These include phosphate binders, which are commonly used to treat CKD complications, as well as antibiotics (e.g., cephalosporins), anticonvulsants, statins, and anticoagulants, which potentially impair vitamin K metabolism and reduce circulating VKDPs [78,79,80,81]. Notably, vitamin K antagonists such as warfarin (originally used as an anticoagulant rodenticide) were, until recently, the main treatment for patients requiring anticoagulation therapy for conditions like atrial fibrillation, pulmonary embolism, or thrombophilia [82]. These drugs work by inhibiting VKOR, an enzyme necessary for the vitamin K metabolic cycle (Figure 2) [83]. However, this vitamin K blockade not only provides anticoagulant benefits but also promotes accelerated VC and calciphylaxis, as observed both experimentally and clinically [84,85,86]. Fortunately, studies have shown that vitamin K supplementation following exposure to antagonists can reverse arterial mineralization [87,88,89].

Finally, the predominant uremic environment in CKD patients is characterized by the presence of uremic toxins, waste products, and impaired metabolome that appear to interfere with the activity of vitamin K recycling molecules and enzymes [75,90]. While the exact mechanisms are still being studied, uremia may regulate vitamin K metabolism by inducing post-transcriptional modifications and disrupting RNA expression, leading to abnormal enzyme conversion in tissues [91]. Alongside, there are reports of a genetic predisposition to the deranged vitamin K cycle that is common in ESKD patients [92,93,94,95]. All these factors act synergically and result in a marked deficiency of vitamin K in ESKD.

### 3.3. Clinical Consequences of Vitamin K Deficiency

Several large observational studies have shown that vitamin K deficiency is associated with worse CVD outcomes [9]. The Rotterdam Study, a population-based study, followed over 4800 participants over a period of 10 years and found that subjects with suboptimal vitamin K dietary intake and low concentrations of vitamin K2 (menaquinones 4 to 10) had a higher risk of aortic calcification, CVD, and increased all-cause mortality. Interestingly, this study did not find a significant association between vitamin K1 and these clinical outcomes [96]. Similarly, the PREVEND (Prevention of Renal and Vascular End-Stage Disease) study included citizens of Groningen (4275 participants included) and followed them for 8.5 years. The study showed an association between functional vitamin K deficiency and increased all-cause and CVD mortality, in both the general population and the subgroup of participants with CKD [97]. In CKD patients, this was further confirmed by the results of the Third National Health and Nutrition Examination Survey (NHANES III), which reported that patients with lower consumption of vitamin K-rich foods were associated with higher all-cause mortality and CVD mortality after a follow-up period of 13.3 years and a total of 37,408 person-years [98].

The dynamic process of bone remodeling that is impaired in CKD patients, called CKD-mineral and bone disease (CKD-MB), involves vitamin K in the complex interaction of vitamin D, PTH, and fibroblast growth factor 23 (FGF-23) [99]. Several mechanisms have been proposed for the contribution of vitamin K to bone formation [100]. Vitamin K2 could inhibit bone resorption by reducing the concentrations of interleukin-6 and prostaglandin E2 within bone tissue [101]. Vitamin K2 also functions as a necessary cofactor of osteocalcin, an important VKDP and crucial player in bone mineralization. When activated by vitamin K, osteocalcin binds calcium to hydroxyapatite, the main mineral component of bones, leading to bone formation [102,103]. In clinical research, an exploratory analysis of a study that followed patients with ESKD for an average of 5.1 years confirmed that poor functional vitamin K status is associated with inflammation and reduced bone mass. In addition, Vitamin K deficiency was linked to an increased risk of fracture after a kidney transplant [104]. In an Italian study, after a one-year observation of 387 HD patients, researchers concluded that vitamin K2 deficiency was a predictor of aortic calcification and that low vitamin K1 levels were a predictor of vertebral fractures [105]. A similar finding was also observed in a previous study of 68 HD patients, which found an association between depleted vitamin K status and increased fracture risk alongside higher PTH levels [106].

A body of growing evidence is linking deficiency to CVD and bone complications, especially in CKD patients. However, the measurement, function, and supplementation are often underused in adult clinical practice, resulting in millions of patients who are still prescribed vitamin K antagonists [99,107,108]. This could be detrimental to a high-risk population like CKD, where vitamin K deficiency is highly prevalent.

## 4. Studies Examining the Effect of Vitamin K Supplementation in Uremia

Recognizing the depleted vitamin K status in CKD patients and its potential consequences on CV health, replenishing vitamin K reserves appears to be a rational approach. Among the existing options for vitamin K supplementation, currently, most research favors menaquinone-7 (MK-7), a commercially available member of the vitamin K2 family [60,109,110]. MK-7 stands out due to its pharmacological properties: it has the longest chain length, which leads to a significantly longer half-life (approx. 3 days) and optimal bioavailability in humans [77,111]. At the molecular level, MK-7 easily integrates into low-density lipoproteins (LDLs), facilitating transport to extrahepatic tissues, such as vessels and bone tissue [112]. These properties make it superior to other forms of menaquinone (with shorter chains and lower bioavailability) and phylloquinone (vitamin K1), which are concentrated primarily in the liver [113,114]. Based on the potential benefits of MK-7 supplementation on vascular health in CKD patients, several randomized controlled trials (RCTs) have been conducted to investigate its effectiveness [115,116]. These studies examined the effects of MK-7 on various parameters, including PWV (a measure of arterial stiffness), progression of CAC (a marker of cardiovascular risk), and cardiovascular events. Table 1 summarizes the interventional trials with vitamin K supplementation in CKD patients, dialysis patients and kidney transplant recipients.

### 4.1. CKD Populations

In vitro and animal studies have initially shown positive results regarding the ability of vitamin K supplementation to reverse VC by reducing calcium deposition in the aorta, carotid, and coronary arteries in uremic models [85,87,129].

The data for non-dialysis CKD patients remain limited, despite the common occurrence of vitamin K deficiency in this population. To date, only two small, single-center RCTs have been conducted. In the first study by Kurnatowska et al. (2015), 42 non-dialysis patients (CKD stage 3–5) were administered either 90 µg of vitamin K2 (MK-7) in combination with vitamin D or vitamin D alone for 270 days. Compared to the control group, in the patients who received MK-7, the mean carotid intima-media thickness (CIMT) was significantly lower, measured by CT scan. However, the CAC score did not reach statistical significance (*p* = 0.07). Among the limitations of the study are the small amounts of vitamin K2 received by the subjects, the small number of participants, and the high variability between the patients in CAC that were recorded prior to treatment [117].

The second RCT (K4Kidneys) was conducted in 159 CKD patients and compared 400 µg of oral MK-7 with a placebo over a 12-month period. In this study, no significant differences were found between groups in PWV, augmentation index, or aortic calcification score measured by plain radiographs [118]. Notably, the short follow-up duration and the use of X-ray technology, which may not be sensitive enough to detect early changes in calcification, may have limited the ability to detect the potential benefits of MK-7 supplementation. Additionally, the study subjects were relatively young and did not have high rates of vitamin K deficiency, having only moderate decline before treatment. In summary, data on CKD patients not requiring dialysis are limited to just two single-center trials with a relatively small number of participants. These limitations restrict the generalizability of the findings.

### 4.2. Dialysis Patients

In recent years, several studies have been conducted on dialysis patients that highlight the potential benefits of vitamin K supplementation on cardiovascular outcomes. A total of seven RCTs were published in just 4 years.

A single-center RCT in Greece involved 52 HD patients, receiving either 200 µg of vitamin K2 (MK-7) or placebo daily for one year. Aortic calcification was assessed using the Agatston score on CT scans, and no significant difference was found between groups. Limitations include the low dose of vitamin K, which was not enough to completely restore the vitamin K levels of the participants (ucMGP was lowered by 45%), the high dropout rate (approx. 50%), and the short follow-up period [119].

The Valkyrie study examined a different approach. A total of 132 HD patients with atrial fibrillation were randomized to receive a vitamin K antagonist, rivaroxaban alone, or rivaroxaban plus 2000 µg of MK-7 three times weekly. As part of the study, patients were followed for 18 months, where cardiac calcium levels, thoracic aortic calcium levels, and PWV were measured. VC values, cardiovascular events, and all-cause mortality were similar across the groups [120]. However, the dp-ucMGP levels of the MK-7 group were persistently high (average 850 pmol/L) even after 18 months of supplementation, indicating inadequate supplementation, and several patients were lost during follow-up, with most of them being of advanced age (mean age: 79.6 years), where arteries are more or less irreversibly mummified by calcium deposition. Interestingly, when the follow-up period was extended by an additional 18 months, the rivaroxaban and rivaroxaban plus vitamin K groups showed lower risk rates for cardiovascular events compared to the warfarin group [130].

The RenaKvit trial enrolled 48 HD and PD patients, which were randomized to 360 µg of MK-7 or placebo. This study had the longest follow-up (2 years). Despite substantial improvements in arterial calcification observed in the vitamin K2 arm, VC was not significantly affected based on measurements of carotid-femoral PWV and calcification scores of the aorta and cardiac valves (Agatston scores) [121]. The effectiveness of the treatment was likely compromised by the fact that almost 90% of participants in the active group continued to receive non-calcium phosphate binders and, as a result, only a modest decrease (40%) in dp-ucMGP levels was documented.

Currently, the largest study, “Trevasc-HDK” involved 138 dialysis patients randomized to receive 360 µg of MK-7 three times weekly or placebo. At 18 months, no significant differences were documented in the primary endpoint (CAC score) or secondary endpoints (aortic valve calcification, carotid-femoral PWV, aortic augmentation index, and cardiovascular events) [122]. Again, the vitamin K2 group showed numerically better results on all VC endpoints, similar to the previous studies, while no statistical significance was reached. Due to the low dose of vitamin K2 administered, dp-ucMGP levels remained expectedly high in both the control and treatment groups at the end of the study (2986 vs. 2500 pmol/L). Furthermore, the authors reported that a minimum number of 170 patients would be required to detect a statistical difference, but this was not feasible due to the relatively high dropout rate. Finally, an open multicenter RCT from Thailand enrolled 96 HD patients, half of whom received 360 µg of MK-7 daily and the other half a placebo. While carotid-femoral PWV (the primary endpoint) showed no significant difference between groups at six months, diabetics in the MK-7 group showed significantly reduced PWV. In addition, all patients who consumed vitamin K2 showed a lower rate of arterial stiffness progression compared to controls [123]. The last two studies only included participants from Asian countries, so the generalizability of the findings to other ethnicities is unclear.

Two recent studies examined the supplementation with vitamin K1 (phylloquinone) instead of vitamin K2. The “iPACK-HD” is a multicenter placebo-controlled trial, which compared 10 mg of phylloquinone thrice weekly to the placebo in 86 HD patients. Vitamin K status improved significantly, but the CAC score showed no difference in terms of absolute score or progression after 12 months. Notably, this was a feasibility study, designed to assess the practicality and effectiveness of conducting a larger-scale trial. In addition, there was an age discrepancy between groups, with a higher proportion of younger patients (48–60 years old) in the placebo group compared to the vitamin K group (almost all above 60 years old) who showed substantial and potentially irreversible VC at baseline [125]. The second vitamin K1 study, the “VitaVasK” trial followed 40 patients for 18 months, and the average thoracic aortic and the CAC were 56% and 68% lower, respectively, in the vitamin K1 group compared to placebo [124]. These results suggest a slower calcification progression rate in major arteries with phylloquinone supplementation.

In conclusion, despite the growing number of studies in recent years, several factors make it difficult to generalize their findings. These include discrepancies in vitamin K dosing, heterogeneity in study design, the use of different endpoints among the studies, and, crucially, diverse methods for measuring VC. These observed inconsistencies make it difficult to draw clear-cut conclusions about whether vitamin K administration improves vascular health in dialysis patients.

### 4.3. Kidney Transplant Recipients

In kidney transplant recipients (KTRs), supplementation of vitamin K seems to have a more clear-cut beneficial effect. A single-center trial involving 60 patients reported that daily supplementation with 360 µg of MK-7 significantly reduced both dp-ucMGP levels and carotid-femoral PWV values by 14.2% (*p* < 0.001) within an 8-week period. Significantly, the greatest improvement in arterial stiffness was observed in patients who were vitamin K deficient at baseline [126]. However, the “ViKTORIES” trial, a double-blind RCT conducted in the same year, tested an experimental synthetic vitamin K3 molecule (menadiol diphosphate) on 45 patients. The authors reported that the treatment group did not benefit when compared to the placebo group in terms of vascular calcification (CAC score) or vascular stiffness (ascending aortic distensibility) [127]. A possible explanation is that the majority of patients included had severe arteriosclerosis before the procedure and, in addition, it was a small sample (only 72 completed the study) and a short follow-up period (12 months).

An important shortcoming of this study was that being the first to test menadiol diphosphate supplementation in KRTs, there were no prior studies determining the dose and the duration of the vitamin K therapy, whereas the authors stated as a limitation the fact that dp-ucMGP values below 900 pmol/L were not accurately quantified and therefore, vitamin K status was not identified or stratified in these values. Finally, at baseline, the two patient groups differed significantly regarding traditional CV risk factors favoring the placebo group; the active group had significantly increased albuminuria and a higher prevalence of diabetes and CVD.

Recently, Sun et al. conducted a meta-analysis to further investigate the role of vitamin K in KTRs, given the scarcity of data in this population. After analyzing seven studies encompassing 1101 patients, the researchers concluded that vitamin K positively impacted patient survival, reducing all-cause mortality, and contributed to improved kidney function by increasing the mean GFR [128]. This meta-analysis had low publication bias and relatively adequate sensitivity analysis, factors improving the robustness of reported results. Moreover, the majority of data was derived from a large, well-designed study by the Transplant Lines investigators and the Dutch group, who measured circulating dp-ucMGP in 578 KTRs and separately in 124 KTRs prior and 90 days following the KT procedure. The authors found a steep (mean −643 pmol/L) decline in dp-ucMGP after KT and every 10 mL/min/1.73 m^2^ increase in eGFR was associated with a 14.0% reduction in dp-ucMGP levels [131].

These findings support the adoption of biomarkers that are independent of kidney function (e.g., dp-ucMGP) for the evaluation of vitamin K status in both everyday clinical practice and experimental research.

### 4.4. Critical Assessment of Interventional Studies

Up to date, the results reported by the existing trials on vitamin K supplementation in CKD patients remain controversial. However, before rejecting the idea of vitamin K supplementation, a critical assessment of the quality, the shortcomings, and the limitations of these data should be discussed.

The studies presented above exhibit significant heterogeneity. This variability extends to the difference in the degree of VC at baseline, the vitamin K types used (phylloquinone, menaquinone, menadiol), the dosage, and the frequency of administration. Moreover, the short follow-up durations (on average below 1.5 years) in most of the trials pose an important limitation [116]. Most studies enrolled fewer than one hundred patients in both arms of the randomized trial, which, in combination with a relatively high dropout rate, lowers the statistical power [132]. Additionally, the majority of participants in these studies were elderly patients or those already exhibiting advanced arterial calcifications, indicating late-stage vascular calcification, where reversal of damage may be practically impossible [133,134]. Finally, due to blind randomization in certain cases, the vitamin K group had a heavier CV burden or had optimal vitamin K levels at baseline, thus jeopardizing the research question and therapeutic target of the study.

These concerns were recently underscored in a meta-analysis conducted by Andrian and colleagues. Encompassing 830 HD patients from 11 RCTs, their analysis also concluded that there was no improvement observed in terms of mortality and VC following vitamin K administration. However, upon scrutinizing the data, it becomes evident that there is high variability in vitamin K dosing (100 to 2000 µg), population heterogeneity (including both pediatric and elderly patients), and disparate endpoints being assessed, while half of the studies do not include mortality or VC on their measurements, limiting their applicability to real-world clinical settings [115]. Conversely, a meta-analysis of 13 controlled clinical trials (n = 2162) and 14 longitudinal studies (n = 10,726) showed that Vitamin K supplementation was accompanied by a significant 9.1% (95% CI −17.7 to −0.5) decrease in the degree of VC and a non-significant improvement in arterial stiffness, due to a 44.7% (95% CI −65.1 to −24.3) decrease in plasma dp-ucMGP levels. Moreover, longitudinal data with long follow-up periods (median 7.8 years) showed that circulating VKDPs were strong predictors of CVD or death (HR 0.45, 95% CI 0.07 to 0.83) [135].

Among these factors, the dosage of vitamin K is a neglected but crucial factor. Although there is no established daily dosage recommendation for vitamin K in kidney disease [68], the significant vitamin K deficiency in these patients is well documented due to factors that impede absorption, distribution, and bioavailability [74,136]. Existing studies have typically employed a dosage range of 90 to 360 µg of MK-7 per day. Interestingly, the 360 μg upper cut-off limit has been chosen quite arbitrarily from the 360 days of the year. The exact dose of vitamin K2 needed to achieve optimal vitamin K status in HD was first investigated in two separate studies by Westenfeld et al. and Caluwe et al. [137,138]. Westenfeld et al., tested lower doses of 45, 135, or 360 µg/d for six weeks, achieving reductions in dp-ucMGP levels of 77% and 93% with doses of 150 µg and 360 µg, respectively. However, even the highest dose of 360 µg daily did not normalize dp-ucMGP values [138]. Similarly, Caluwe et al. evaluated higher doses of 360, 720, and 1080 µg three times a week, reporting substantial reductions in dp-ucMGP levels as well, yet many patients still exhibited abnormal levels [137]. It should be emphasized that the use of MK-7 or phylloquinone, even at high doses, has not been associated with major adverse events, making it a safe and well-tolerated therapy [139,140]. Consequently, the justification of these dosages requires re-evaluation, as they may not be adequate to ensure vitamin K sufficiency in CKD patients, and higher doses are likely required for meaningful effects to occur.

Additionally, sub-analyses of certain studies have revealed that the beneficial effects of vitamin K supplementation reached statistical significance in patients who were identified as having vitamin K deficiency prior to the intervention [7,126,127]. While depleted levels of vitamin K are common among CKD patients, it is important to recognize that not all individuals with CKD may exhibit such deficiencies [141]. Therefore, it is crucial to identify specific subgroups of CKD patients who may derive the most benefit from this treatment approach.

In this direction, evidence suggests that diabetic patients could particularly benefit from vitamin K supplementation, as reported by both observational and interventional studies in patients with diabetic nephropathy [24,123]. Another study explored a different approach by studying genetic variabilities in diabetic patients, finding a genetic polymorphism (T-138c) of the MGP gene that is linked with CIMT and serves as a robust and independent predictor of all-cause mortality in patients with diabetic nephropathy [92,142]. Therefore, larger studies, including a greater sampling of CKD patients, are needed to better understand the specific populations that may benefit most from vitamin K supplementation. For instance, the underrepresentation of peritoneal dialysis (PD) patients, with fewer than twenty individuals included in the totals of current data [115], raises questions about the potential effects of vitamin K on this subgroup.

## 5. Future Directions

### 5.1. Ongoing Trials

Despite the growing body of research in recent years, it will be crucial to gain further insights into vitamin K supplementation and its effects on VC and CVD in CKD patients. Studies conducted to date have raised concerns about whether vitamin K administration can effectively reduce the increased cardiovascular risk associated with CKD. Fortunately, seven additional trials are currently underway that may help better understand the potential value of vitamin K supplementation in this patient population.

The “VitK-CUA” trial (NCT02278692) investigates whether 10 mg vitamin K1 (VK1) thrice weekly improves VC and calciphylaxis in CKD patients. Meanwhile, the “Vita-K ‘n’ CKD” (NCT03311321) is an 8-week, placebo-controlled RCT study examining whether daily vitamin K2 supplementation (360 µg MK-7 a day) enhances endothelial function and improves arterial stiffness in HD patients. The “VIKIPEDIA” study (NCT04900610) marks the first trial in PD patients, evaluating the effect of higher doses of vitamin K (1 mg MK-7 daily) on arterial stiffness by measuring changes in PWV and ambulatory blood pressure [143]. The UCASAL-VITK trial (NCT04539418) examines the effect of high doses of MK-7 as well (1000 µg three times weekly) administered intravenously after each dialysis session on CIMT in HD patients. Additionally, a Japanese study (UMIN000011490) tests a synthetic analog of MK-4 (Menatetrone) on dialysis patients. Researchers will assess changes in abdominal aortic calcification 12 and 24 months after daily administration of 45 mg of the drug [144]. An Egyptian trial (NCT04477811) compares the effects of vitamin K1, vitamin K2, and placebo in HD patients, focusing on changes in dp-ucMGP levels. Finally, the Canadian “VISTA” trial (NCT02324686) investigates whether 400 µg of vitamin K1 three times weekly helps control the international normalized ratio (INR) in HD patients with atrial fibrillation.

### 5.2. Novel Biomarkers

The contraindicatory results of existing data raise the clinical question of whether it is feasible to reverse the calcification process in patients with already advanced vascular damage. Earlier, timely identification of increased risk for VC will allow for prompt treatment and therapeutic decisions. In this direction, investigators are exploring novel biomarkers that detect early signs of VC. T50 and the Biohybrid Assay have emerged as two such promising examples of biomarkers.

Transformation time (T50), also called the serum calcification propensity test, is a functional test that measures the inherent ability of a patient’s serum to inhibit the precipitation of calcium and phosphate. By artificially increasing the calcium and phosphate concentration in the serum, the conversion of spherical colloidal primary calciprotein particles (CPPs) into secondary CPPs is triggered [145]. CPPs are essentially colloidal particles formed in the serum in place of crystalline hydroxyapatite [146]. This assay measures the time-resolved changes in nephelometry during the conversion of primary CPPs to secondary CPPs [145]. A meta-analysis by Pluquet et al. included 57 publications and 23 clinical studies on CKD, dialysis patients, and kidney transplant recipients [147]. They concluded that T50 associates with CVD, mortality, and graft loss in this population. The important limitation of this biomarker is that it depends solely on a chemical reaction without assessing the influence of the vascular endothelial environment.

The BioHybrid assay, developed by Schurgers et al., may offer several advantages over T50 in identifying calcification proclivity. The BioHybrid assay, as its name suggests, is a cell-based assay that uses human vascular smooth muscle cells (hVSMCs), taken from non-atherosclerotic abdominal aortas and then cultured in vitro [148]. After inducing the calcification process and adding human serum, scientists can measure real-time calcification development within the assay, unlike T50, which measures a delayed chemical reaction in serum. The BioHybrid was found to be more sensitive in discerning the calcification risk by evaluating the total circulating components that can either inhibit or promote calcifications (serum functional anti-calcifying and buffering capacity, microcell calcification), including the uremic toxins in CKD patients. For instance, the BioHybrid assay could discern differences in measurements between post-dialysis and control serum. On the contrary, this discrimination was not performed by T50, which showed no difference in the human serum of controls and that of HD patients after the procedure [149]. However, it is important to note that this method has yet to be applied in real-world clinical settings.

To summarize, innovative biomarkers for VC, like T50 and the BioHybrid assay, could serve as valuable tools to detect high-risk patients for advanced stages of VC early on and also as potential therapeutic targets of novel agents that aim to prevent or reverse the VC process. Further research is essential to validate their value in clinical settings.

### 5.3. Suggestion for the Design of Future RCTs Examining the Effect of Vitamin K Supplementation on VC and CV Outcomes in Uremic Patients

Since there were several methodological flaws and limitations in existing studies, herein we provide suggestions to improve the design of future RCTs examining the effect of vitamin K supplementation on CV outcomes in uremic patients. The ideal RCT should have a large sample size, (taking into consideration a high dropout rate), a long follow-up period (over 18 months), and in the enrolled population, elderly patients whose arteries are already irreversibly calcified should be excluded. Both vitamin K1 and K2 could be administered; however, the dosage should be carefully selected. We know from dose-dependent studies that even 460 μg/day of K2 are under therapeutic in dialysis patients, and on the other hand, even large doses of 20 mg of K1 and 2000 mg/kg ΜΚ-7 per body weight are safe, well-tolerated, and not toxic. Therefore, in dialysis patients, we recommend doses of MK-7 > 500 μg/day. Notably, the ongoing VIKIPEDIA trial examines the effect of 1000 μg of MK-7/day in PD patients. An important but overlooked aspect is the blind randomization of existing RCTs, which in some cases resulted in treating vitamin K patients with normal vitamin K status. We recommend that the categorization of the K and the control groups should not be performed blindly but based on vitamin K status, which should be assessed by circulating dp-ucMGP when we focus on the arterial wall and by PIVKA-II when we are more interested in hepatic vitamin K status. To ensure that vitamin K can exert its biological effects, we need to first, at baseline, correct potential vitamin D and magnesium deficiencies, and terminate phosphate binders (such as sevelamer) that might undesirably bind and reduce vitamin K bioavailability. However, we should also manage all other well-known factors promoting VC, such as hyperphosphatemia, hyperparathyroidism, etc., because we believe that the management of VC in CKD patients needs a multifractional approach where vitamin K might be a cornerstone but surely not a sole holy-grail therapy. The adopted endpoints should include a variety of established surrogate markers of VC and stiffness (such as CAC, PWV, IMT) but also novel, experimental ones (such as T50 and the Biohybrid assay) and on top of that, clinically hard endpoints, including deterioration of kidney function, mortality, initiation of dialysis, and CV events. Notably, vitamin K has been suggested to exert pleiotropic effects on muscle/joint pain, headaches, cognitive function, and bone metabolism as well. The VIKIPEDIA trial will examine all these endpoints. We need more and well-designed, large RCTs, to elucidate the area of vitamin K supplementation in CKD and dialysis patients.

## 6. Conclusions

In the era of widespread use of multivitamins contained in over-the-counter products, vitamin K supplements with their generally safe profile remain underutilized, despite documented high rates of vitamin K deficiency in CKD patients. While a growing number of clinical trials have examined the encouraging results of preclinical studies, these studies cannot currently justify the widespread adoption of vitamin K supplementation in everyday clinical care as a VC treatment strategy. We now stand upon a research paradox; although nephrologists tended to prescribe warfarin to dialysis patients, which was first launched as a rat poison, researchers hesitate to conduct trials administering vitamin K in doses similar to those pediatricians give to newborn babies.

Before we definitively conclude that vitamin K does not offer significant benefits in CVD, several aspects and limitations of existing studies must be considered. Future studies facilitating larger patient populations, longer follow-up periods, and especially higher vitamin K doses are crucial. Furthermore, identifying specific subgroups of CKD patients who may benefit most from nutritional supplementation could significantly improve therapeutic outcomes.

## Figures and Tables

**Table 1:** Interventional trials with vitamin K supplementation in CKD patients, dialysis patients, and kidney transplant recipients.

Name of the Study	Year	N	Vitamin K	Dose	Duration	Groups	Result	Limitations	Strengths
Non-dialysis CKD Patients									
Kurnatowska et al. [117]	2015	42	MK-7	90 μg/day	9 months	Vitamin K + D/Vitamin D	Reduced progress of CIMT/CACS towards benefit	-small sample -short follow-up -low dose	-one of the first RCTs with vitamin K in CKD -one of the first RCTs to examine the synergy between vitamins K + D -extended analysis excluding 4 patients with markedly increased calcification scores
K4Kidneys [118]	2020	159	MK-7	400 μg/day	12 months	Vitamin K/Placebo	No effect on PWV or VC	-mean age of enrolled patients was lower than that of typical CKD 3b-4 patients -the study population did not exhibit severe vitamin K depletion	-large sample -the first large RCT in CKD -the RCT with the highest MK-7 dosage
Dialysis Patients									
Oikonomaki et al. [119]	2019	52	MK-7	200 μg/day	12 months	Single Group	No effect on Agatston scores in aortic calcification	-low dose of vitamin K -ucMGP was only 45% reduced -50% dropout rate -short follow-up period -small sample	-the first RCT in HD
Valkyrie [120]	2020	132	MK-7	200 μg × 3/week	18 months	Warfarin/Rivaroxaban/Rivaroxaban + MK-7	No effect on vascular stiffness or cardiac valve calcification	-dp-ucMGP levels of the MK-7 group remained high -low dose -short follow-up -mean age of patients: 79.6 years -many lost to follow-up	-large sample -when follow-up was extended by an additional 18 months, rivaroxaban and rivaroxaban plus vitamin K, showed lower risk rates for CV events compared to the warfarin
RenaKvit [121]	2021	52	MK-7	360 μg/day	24 months	Vitamin K/Placebo	No effect on carotid-femoral PWV or VC	-small size -low dose -mixed HD + PD -90% of the active group received non-calcium phosphate binders -only 40% decrease in dp-ucMGP -high dropout (only 21 patients completed the study)	-long follow-up
Trevasc-HDK [122]	2023	138	MK-7	360 μg × 3/week	18 months	Vitamin K/Placebo	No effect on CAC score or carotid-femoral PWV or cardiac valve calcification	-low dose -dpucMGP remained high in the active group -high dropout rate -underpowered study (170 patients were needed) -only Asian population	-large sample -many CV endpoints
Naiyarakseree et al. [123]	2023	96	MK-7	360 μg/day	6 months	Vitamin K/Placebo	No effect on carotid-femoral PWV	-small sample -short follow-up -low dose -only Asian population	-subgroup analysis in diabetics showed significant effect of vitamin K on PWV
VitaVasK [124]	2022	40	K1	5 mg × 3/week	18 months	Vitamin K/Placebo	TAC score 56% reduced and CAC score 68% lower	-small sample	-gold standard endpoints
iPACK-HD [125]	2023	86	K1	10 mg × 3/week	12 months	Vitamin K/Placebo	No effect on CAC score	-feasibility study -not designed to detect outcomes -age discrepancy between groups in favor of the placebo group -the K group patients had substantial and potentially irreversible VC at baseline	-showed that K1 supplementation was safe and well tolerated in HD patients
Kidney Transplant Recipients									
KING [126]	2017	60	MK-7	360 μg/day	2 months	Single Group	14.2% reduction in PWV	-short follow-up -endpoint was a surrogate marker of vascular stiffness and not a clinical hard outcome	-showed that vitamin K ad effect in the vitamin K deficient patients
ViKTORIES [127]	2021	90	Menadiol Diphosphate	5 mg × 3/week	12 months	Vitamin K/Placebo	No effect on VC or vascular stiffness	-the dose and duration of follow-up for menadiol was not known -short follow-up -small sample -high dropout rate -the majority of patients had severe arteriosclerosis at baseline -dp-ucMGP < 900 pmol/L was not accurately quantified -the active group had significantly increased albuminuria and higher prevalence of diabetes and CVD at baseline	-the first to test menadiol diphosphate supplementation in KRTs,
Meta-analysis Studies									
Andrian et al. [115]	2023	830	K1/K2	variable	6 weeks to 24 months	Both adult and pediatric HD patients	Non-significant trend in reducing calcification scores	-high variability in vitamin K dosing, population, and endpoints −50% of studies do not include mortality or VC	-large sample/many studies
Sun et al. [128]	2023	1101	not specified	variable	-	KTR	Reduced all-cause mortality, improvement in GFR	-Meta-analysis and not RCT -not specified form/dose of vitamin K	-low publication bias -adequate sensitivity analysis -Quantified the effect of kidney function on vitamin K status

Abbreviations; CAC: Coronary Artery Calcification, CKD: Chronic Kidney Disease, CIMT: Carotid Intima-Media Thickness, GFR: Glomerular Filtration Rate, HD: Hemodialysis, KTR: Kidney Transplant Recipients, MK-7: Menaquinone-7, PWV: Pulse Wave Velocity, VC: Vascular Calcification, TAC: Thoracic Aorta Calcification.

## Data Availability

All data are available within the manuscript.
